# WHO global research priorities for antimicrobial resistance in human health

**DOI:** 10.1016/S2666-5247(24)00134-4

**Published:** 2024-11

**Authors:** Silvia Bertagnolio, Zlatina Dobreva, Chad M Centner, Ioana Diana Olaru, Daniele Donà, Stefano Burzo, Benedikt D Huttner, Antoine Chaillon, Nebiat Gebreselassie, Teodora Wi, Mateusz Hasso-Agopsowicz, Benedetta Allegranzi, Hatim Sati, Verica Ivanovska, Kavita U Kothari, Hanan H Balkhy, Alessandro Cassini, Raph L Hamers, Kitty Van Weezenbeek, WHO Research Agenda for AMR in Human Health Collaborators, David Aanensen, Alexandre Alanio, Ana Alastruey-Izquierdo, Tinsae Alemayehu, Majdi Al-Hasan, Karel Allegaert, Amal Saif Al-Maani, Jameela Al-Salman, Abeer Nizar Alshukairi, Afreenish Amir, Tanya Applegate, George F Araj, Marlen Arce Villalobos, Christine Årdal, Diane Ashiru-Oredope, Elizabeth A Ashley, François-Xavier Babin, Laura H Bachmann, Till Bachmann, Kate Susan Baker, Manica Balasegaram, Colleen Bamford, Fernando Baquero, Laura Isabel Barcelona, Quique Bassat, Matteo Bassetti, Sulagna Basu, Justin Beardsley, Grey Benoit Vásquez, James A Berkley, Anuj K Bhatnagar, Julia Bielicki, Julie Bines, Felix Bongomin, Robert A Bonomo, John S Bradley, Catriona Bradshaw, Ana Brett, Adrian Brink, Colin Brown, Jeremy Brown, Kirsty Buising, Carolee Carson, Anna Cristina Carvalho, Elio Castagnola, Marco Cavaleri, Michele Cecchini, Chishala Chabala, Richard E Chaisson, Arunaloke Chakrabarti, Clare Chandler, Sujith John Chandy, Esmita Charani, Lisa Chen, Francesca Chiara, Anuradha Chowdhary, Arlene Chua, Pem Chuki, Doo Ryeon Chun, Gavin Churchyard, Daniela Cirillo, Lauren Clack, Susan E Coffin, Jennifer Cohn, Michelle Cole, John Conly, Ben Cooper, Alejandra Corso, Sara E Cosgrove, Helen Cox, Charles L Daley, Saffiatou Darboe, Tom Darton, Gerry Davies, Viviana de Egea, Amela Dedeić-Ljubović, Miranda Deeves, Claudia Denkinger, Jo-Anne R Dillon, Angela Dramowski, Brian Eley, Susanna Maria Roberta Esposito, Sabiha Y Essack, Helmia Farida, Joveria Farooqi, Nicholas Feasey, Cecilia Ferreyra, Helen Fifer, Heather Finlayson, Mike Frick, Ana Cristina Gales, Luisa Galli, Sumanth Gandra, Jeffrey S Gerber, Christian Giske, Bruce Gordon, Nelesh Govender, Nathalie Guessennd, Ibrehima Guindo, Elmira Gurbanova, Amanda Gwee, Ferry Hagen, Stephan Harbarth, John Haze, Jutta Heim, Rene Hendriksen, Robert Simon Heyderman, Kathryn Elizabeth Holt, Martin Hönigl, Edward W Hook, William Hope, Heidi Hopkins, Gwenda Hughes, Ghada Ismail, Mohammad Iqbal Issack, Jan Jacobs, Dušan Jasovský, Fyeza Jehan, Antonieta Jimenez Pearson, Makoto Jones, Mohan P Joshi, Arti Kapil, Samuel Kariuki, Abhilasha Karkey, Gregory L Kearns, Karen Helena Keddy, Nina Khanna, Akiko Kitamura, Kaija-Leena Kolho, Dimitrios P Kontoyiannis, Anita Kotwani, Roman S Kozlov, Katharina Kranzer, Ranmini Kularatne, Monica M Lahra, Bradley J Langford, Rafael Laniado-Laborin, Joakim Larsson, Cornelia Lass-Flörl, Kirsty Le Doare, Hyukmin Lee, Fernanda Lessa, Anna S Levin, Direk Limmathurotsakul, Nilton Lincopan, Andrea Lo Vecchio, Rakesh Lodha, Mark Loeb, Yves Longtin, David Chien Lye, Asif Mujtaba Mahmud, Célia Manaia, Lenore Manderson, Ivana Mareković, Kalisvar Marimuthu, Irene Martin, Tapfumanei Mashe, Zeng Mei, Jacques F Meis, Flávio Augusto Lyra Tavares De Melo, Marc Mendelson, Angelica Espinosa Miranda, David Moore, Chantal Morel, Nyambura Moremi, Maria Luisa Moro, Francis Moussy, Stephen Mshana, Arno Mueller, Francis J Ndow, Mark Nicol, Andrew Nunn, Stephen Obaro, Christina W Obiero, Iruka N Okeke, Uduak Okomo, Tochi J Okwor, Rita Oladele, Sylvia Omulo, Pascale Ondoa, Juana Medarda Ortellado de Canese, Luis Ostrosky-Zeichner, Maria Clara Padoveze, Madhukar Pai, Benjamin Park, Julian Parkhill, Christopher M Parry, Rosanna Peeling, Luísa Maria Sobreira Vieira Peixe, Olga Perovic, Melinda M Pettigrew, Nicola Principi, Céline Pulcini, Nelly Puspandari, Timothy Rawson, Denasha Lavanya Reddy, Kessendri Reddy, Paulo Redner, Juan Luis Rodríguez Tudela, Jesús Rodríguez-Baño, Susan Rogers Van Katwyk, Emmanuel Roilides, Christine Rollier, Leslie Rollock, Jean-Baptiste Ronat, Etienne Ruppe, Manish Sadarangani, David Salisbury, Mounerou Salou, Luc Hervé Samison, Maurizio Sanguinetti, Massimo Sartelli, Natalie Schellack, Jeroen Schouten, Mitchell J Schwaber, Jeremiah Seni, Abiola Senok, William M Shafer, Sadia Shakoor, Donald Sheppard, Jong-Hee Shin, Sonia Sia, Dawn Sievert, Ishwar Singh, Rupak Singla, Robert Leo Skov, Olusegun O Soge, Rosanne Sprute, Arjun Srinivasan, Subasree Srinivasan, Arnfinn Sundsfjord, Evelina Tacconelli, Sabira Tahseen, Viroj Tangcharoensathien, Thomas Tängdén, Karin Thursky, Guy Thwaites, Renata Tigulini de Souza Peral, Deborah Tong, Hafsah Deepa Tootla, Constantinos Tsioutis, Katy M Turner, Paul Turner, Shaheed Vally Omar, Wendy WJ van de Sande, Susan van den Hof, Rogier van Doorn, Balaji Veeraraghavan, Paul Verweij, Retno Wahyuningsih, Hui Wang, Adilia Warris, Hillard Weinstock, Evelyn Wesangula, David Whiley, Peter J White, Phoebe Williams, Yonghong Xiao, Martin Yagui Moscoso, Hsu Li Yang, Sachiyo Yoshida, Yunsong Yu, Dorota Żabicka, Matteo Zignol, Igor Rudan

**Affiliations:** n University of Oxford, United Kingdom; o Institut Pasteur, Université Paris Cité, National Reference Center for Invasive Mycoses and Antifungals, Translational Mycology research group, Mycology Department, Paris, France; Laboratoire de parasitologie-mycologie, AP-HP, Hôpital Saint-Louis, Paris, France; p Instituto de Salud Carlos III, Spain; q St. Paul's Hospital Millennium Medical College, Addis Ababa, Ethiopia and American medical center, Addis Ababa, Ethiopia; r University of South Carolina School of Medicine; Prisma Health-Midlands, Columbia, South Carolina, United States of America; s KU Leuven, Belgium; t Ministry of Health, Oman; u Salmaniya Medical Complex, Bahrain; v Alfaisal University in Riyadh, Saudi Arabia; Previously King Faisal Specialist Hospital and Research Center in Jeddah, Saudi Arabia; w National Institute of Health, Pakistan; x Kirby Institute, UNSW Sydney, Australia; y American University of Beirut - Medical Center, Lebanon; z Ministerio de Salud, Costa Rica; aa Norwegian Institute of Public Health, Norway; bb UK Health Security Agency, United Kingdom; cc Lao-Oxford-Mahosot Hospital-Wellcome Trust Research Unit (LOMWRU), Mahosot Hospital, Vientiane, Lao PDR; Centre for Tropical Medicine & Global Health, Nuffield Department of Medicine, University of Oxford, Oxford, UK; dd Fondation Mérieux, France; ee Centers for Disease Control and Prevention, United States of America; ff Centre for Inflammation Research, Institute for Regeneration and Repair, The University of Edinburgh, Edinburgh; gg Department of Genetics, University of Cambridge, Downing Place, Cambridge, United Kingdom; hh Global Antibiotic R&D Partnership (GARDP); ii Pathcare, South Africa; jj Ramón y Cajal Institute for Health Research, Centro de Investigación Biomédica en Red de Epidemiología y Salud Pública (CIBERESP), Spain; kk Ministerio de Salud de la Nación, Argentina; ll Barcelona Institute for Global Health (ISGlobal), Spain; mm University of Genoa, Italy; nn ICMR-National Institute of Cholera and Enteric Diseases, Kolkata, India; oo University of Sydney, Australia; pp Dirección de Epidemiología, Ministerio de Salud Pública y Asistencia Social, Dominican Republic; qq KEMRI/Wellcome Trust Research Programme, Kilifi, Kenya; Centre for Tropical Medicine & Global Health, University of Oxford, UK; rr Rajan Babu Institute for Pulmonary Medicine and Tuberculosis, India; ss St George's University of London, United Kingdom; University of Basel Children's Hospital, Switzerland; tt The University of Melbourne; Murdoch Children's Research Institute; Department of Gastroenterology and Clinical Nutrition, The Royal Children's Hospital; uu Gulu University, Uganda; vv Cleveland VA Medical Center, Case Western Reserve University, United States of America; ww University of California, San Diego, School of Medicine; xx Monash University, Australia; yy Clínica Universitária de Pediatria, Faculdade de Medicina, Universidade de Coimbra, Portugal; zz University of Cape Town, South Africa; aaa UK Health Security Agency, United Kingdom; bbb University College London, United Kingdom; ccc Victorian Infectious Diseases Unit, Royal Melbourne Hospital, Australia; Department of Infectious Diseases, University of Melbourne, Australia; ddd Public Health Agency of Canada, Canada; eee Oswaldo Cruz Institute, Fiocruz, Rio de Janeiro, Brazil; fff IRCCS Istituto Giannina Gaslini, Italy; ggg European Medicines Agency, Netherlands; hhh Organisation for Economic Co-operation and Development (OECD), France; iii University of Zambia, Zambia; jjj Johns Hopkins University School of Medicine; Johns Hopkins Center for Tuberculosis Research; Johns Hopkins Center for AIDS Research; kkk Doodhadhari Burfani Hospital and Research Institute, Haridwar, India; lll London School of Hygiene and Tropical Medicine, United Kingdom; mmm Department of Pharmacology & Clinical Pharmacology, Christian Medical College, Vellore, India; nnn Imperial College London, United Kingdom; ooo UCSF Curry International Tuberculosis Center, United States of America; ppp Center for Infectious Disease Research and Policy, United States of America; qqq Medical Mycology Unit, Department of Microbiology, Vallabhbhai Patel Chest Institute, University of Delhi, Delhi; National Reference Laboratory For Antimicrobial Resistance in Fungal Pathogens, Medical Mycology Unit, Department of Microbiology, Patel Chest Institute, University of Delhi, Delhi, India; rrr Médecins Sans Frontières, Switzerland; sss Jigme Dorji Wangchuck National Referral Hospital, Bhutan; ttt Samsung Medical Center, Sungkyunkwan University School of Medicine, Seoul, Republic of Korea; uuu The Aurum Institute, Parktown, South Africa; Department of Medicine, Vanderbilt University, Nashville, TN, US; vvv San Raffaele Scientific Institute, Italy; www University of Zurich, Institute for Implementation Science in Health Care, Zurich, Switzerland; xxx Children’s Hospital of Philadelphia /University of Pennsylvania School of Medicine, United States of America; yyy Global Antibiotic R&D Partnership (GARDP); zzz STI Reference Laboratory, UKHSA, London, UK; aaaa University of Calgary and Alberta Health Services, Canada; bbbb University of Oxford, United Kingdom; cccc Servicio Antimicrobianos - INEI ANLIS Dr Carlos G. Malbrán, Argentina; dddd Johns Hopkins University School of Medicine, United States of America; eeee Institute of Infectious Diseases and Molecular Medicine, Division of Medical Microbiology, University of Cape Town; ffff National Jewish Center, United States of America; gggg MRC Unit The Gambia at the LSHTM; hhhh University of Sheffield, United Kingdom; iiii University of Liverpool, United Kingdom; jjjj Ministry of Health, Paraguay; kkkk Clinical Center of the University Sarajevo, Bosnia and Herzegovina; llll World Health Organization, Geneva, Switzerland; mmmm Division of Infectious Disease and Tropical Medicine, German Center for Infection Research (DZIF) partner site, University Hospital Heidelberg, Germany; nnnn University of Saskatchewan, Canada; oooo Stellenbosch University, South Africa; pppp Department of Paediatrics and Child Health, University of Cape Town, Cape Town, South Africa; qqqq University of Parma, Italy; rrrr Antimicrobial Research Unit, School of Health Sciences, University of KwaZulu-Natal, Durban, South Africa; ssss Universitas Diponegoro, Indonesia; tttt Aga Khan University, Karachi, Pakistan; uuuu School of Medicine, University of St Andrews, St Andrews, UK and Liverpool School of Tropical Medicine, Liverpool, UK; vvvv FIND, Switzerland; wwww UK Health Security Agency, United Kingdom; xxxx Stellenbosch University, South Africa; yyyy Treatment Action Group, United States of America; zzzz Universidade Federal de São Paulo, Brazil; aaaaa Department of Health Science, University of Florence and Infectious Disease Unit, Meyer Children's Hospital, IRCCS, Florence, Italy; bbbbb Washington University School of Medicine in St. Louis, United States of America; ccccc Children's Hospital of Philadelphia, United States of America; ddddd Karolinska Institute, Sweden; eeeee World Health Organization, Geneva, Switzerland; fffff National Institute for Communicable Diseases, South Africa; ggggg Institut Pasteur de Côte d'Ivoire, Ivory Coast; hhhhh National Institute of Public Health, Bamako-Mali, Mali; iiiii Tartu University Clinic, Estonia; jjjjj Department of General Medicine, Royal Children’s Hospital, Antimicrobials Group, Murdoch Children’s Research Institute, Department of Paediatrics, The University of Melbourne; kkkkk Westerdijk Fungal Biodiversity Institute (WI-KNAW), Netherlands; lllll University Hospital Geneva (HUG), Switzerland; mmmmm Ministry of Health, St Vincent & the Grenadines; nnnnn Helmholtz Centre for Infection Research, Switzerland; ooooo Technical University of Denmark, Denmark; ppppp University College London (UCL), United Kingdom; qqqqq London School of Hygiene and Tropical Medicine, United Kingdom; rrrrr Medical University of Graz, Austria; sssss University of Alabama at Birmingham, United States of America; ttttt University of Liverpool, United Kingdom; uuuuu London School of Hygiene and Tropical Medicine, United Kingdom; vvvvv London School of Hygiene and Tropical Medicine, United Kingdom; wwwww Supreme Council for University Hospitals, Egypt; xxxxx Central Health Laboratory, Ministry of Health and Wellness, Mauritius; yyyyy Institute of Tropical Medicine, Antwerp, Belgium; zzzzz Médecins Sans Frontières Access Campaign, Switzerland; aaaaaa The Aga Khan University, Pakistan; bbbbbb National Reference Center of Bacteriology - INCIENSA, Costa Rica; cccccc University of Utah, United States of America; dddddd Medicines, Technologies, and Pharmaceutical Services (MTaPS) Program, Management Sciences for Health, United States of America; eeeeee North DMC Medical College and Hindu Rao Hospital, India; ffffff Director Eastern Africa, Drugs for Neglected Diseases Initiative, Nairobi, Kenya; gggggg Oxford University Clinical Research Unit Nepal, Nepal; hhhhhh Professor of Pediatrics and Medical Education, The Burnett School of Medicine at Texas Christian University, Fort Worth, TX, United States of America; iiiiii Independent Consultant, South Africa; jjjjjj University Hospital Basel, Switzerland; kkkkkk Health, Nutrition and Population Global Practice, World Bank Group, United States of America; llllll Children´s Hospital, University of Helsinki and HUS, Helsinki, Finland; mmmmmm Division of Internal Medicine, Houston, TX, United States of America; nnnnnn Ex-Director professor, Department of Pharmacology, V. P. Chest Institute, University of Delhi, India; oooooo Smolensk State Medical University, Russia; pppppp London School of Hygiene and Tropical Medicine, United Kingdom; qqqqqq Labtests Auckland, New Zealand; rrrrrr WHO Collaborating Centre for ST and AMR, NSWHP Microbiology, The Prince of Wales Hospital, NSW, Australia; ssssss Public Health Ontario, Canada; tttttt Hospital General Tijuana, ISESALUD de Baja California, Mexico; uuuuuu University of Gothenburg, Sweden; vvvvvv Institute of Hygiene and Medical Microbiology European Excellence Center of Medical Mycology (ECMM), Medical University of Innsbruck, Innsbruck, Germany; wwwwww St. George's University of London, United Kingdom; Makerere University, Kampala, Uganda; xxxxxx Severance Hospital, Yonsei University Medical College, Republic of Korea; yyyyyy Centers for Disease Control and Prevention, United States of America; zzzzzz Universidade de São Paulo, Brazil; aaaaaaa Mahidol-Oxford Tropical Medicine Research Unit, Faculty of Tropical Medicine, Bangkok, Thailand; Department of Tropical Hygiene, Faculty of Tropical Medicine, Bangkok, Thailand; Centre for Tropical Medicine and Global Health, Nuffield Department of Medicine, University of Oxford, Oxford, United Kingdom; bbbbbbb Department of Microbiology, Institute of Biomedical Sciences, University of São Paulo, Brazil; ccccccc University of Naples Federico II, Italy; ddddddd All India Institute of Medical Sciences, India; eeeeeee McMaster University, Canada; fffffff Jewish General Hospital, Montreal, Canada; ggggggg National Centre for Infectious Diseases; Tan Tock Seng Hospital; Yong Loo Lin School of Medicine; Lee Kong Chian School of Medicine, Singapore; hhhhhhh Asgar Ali Hospital, Dhaka; Bangladesh Lung Foundation; Formerly at Institute of Epidemiology, Disease Control and Research (IEDCR), Mahakhali, Dhaka, Bangladesh; iiiiiii Universidade Católica Portuguesa, Portugal; jjjjjjj University of the Witwatersrand, South Africa; kkkkkkk University Hospital Centre Zagreb, Department of Clinical Microbiology, Infection Prevention and Control, Zagreb, Croatia; lllllll Tan Tock Seng, Hospital and National Centre for Infectious Diseases, Singapore; mmmmmmm National Microbiology Laboratory, Public Health Agency of Canada, Winnipeg, Canada; nnnnnnn WHO, Ministry of Health and Child Care, Zimbabwe; ooooooo Children's Hospital of Fudan University, National Children's Medical Center, China; ppppppp University of Cologne, Faculty of Medicine and University Hospital Cologne, Department I of Internal Medicine, Center for Integrated Oncology Aachen-Bonn- Cologne-Duesseldorf (CIO ABCD) and Excellence Center for Medical Mycology (ECMM), Cologne, Germany; qqqqqqq Governo do Estado da Paraíba, Brazil; rrrrrrr University of Cape Town, South Africa; sssssss Ministério da Saúde, Brazil; ttttttt Department of Paediatrics and Child Health, Chris Hani Baragwanath Academic Hospital, University of the Witwatersrand, Johannesburg, South Africa; South African Medical Research Council Vaccine and Infectious Diseases Analytics (VIDA) Research Unit, University of the Witwatersrand, Johannesburg, South Africa; uuuuuuu University Hospital Bonn, Institute for Hygiene & Public Health, Germany; vvvvvvv National Public Health Laboratory, Tanzania; wwwwwww Regional Health and Social Agency, Emilia Romagna Region, Italy; xxxxxxx World Health Organization, Geneva, Switzerland; yyyyyyy Catholic University of Health and Allied Sciences, Tanzania; zzzzzzz World Health Organization, Geneva, Switzerland; aaaaaaaa Skin and GU Medicine Clinic, Harare, Zimbabwe; bbbbbbbb University of Western Australia, Australia; cccccccc MRC Clinical Trials Unit at University College London, United Kingdom; dddddddd University of Nebraska Medical Center, United States of America; eeeeeeee Kenya Medical Research Insistute - Wellcome Trust Research Programme, Kenya; ffffffff University of Ibadan, Nigeria; gggggggg MRC Unit The Gambia at London School of Hygiene and Tropical Medicine, Gambia; hhhhhhhh Nigeria Centre for Disease Control and Prevention, Nigeria; iiiiiiii College of Medicine, University of Lagos, Nigeria; jjjjjjjj Washington State University, United States of America; kkkkkkkk African Society for Laboratory Medicine (ASLM), Ethiopia; llllllll Universidad Nacional de Asunción, Paraguay; mmmmmmmm University of Texas Health Science Center at Houston, United States of America; nnnnnnnn School of Nursing, University of São Paulo, Brazil; oooooooo McGill University, Canada; pppppppp Centers for Disease Control and Prevention, United States of America; qqqqqqqq University of Cambridge, United Kingdom; rrrrrrrr Liverpool School of Tropical Medicine, United Kingdom; ssssssss London School of Hygiene and Tropical Medicine, United Kingdom; tttttttt Faculdade de Farmácia, Universidade do Porto, Portugal; uuuuuuuu National Institute for Communicable Diseases a division of NHLS, South Africa; vvvvvvvv Yale School of Public Health, United States of America; wwwwwwww University of Milan, Italy; xxxxxxxx Université de Lorraine et CHRU Nancy, APEMAC et Centre régional en antibiothérapie du Grand Est AntibioEst, Nancy, France; yyyyyyyy Health Policy Agency, Ministry of Health Indonesia, Indonesia; zzzzzzzz Imperial College London, United Kingdom; aaaaaaaaa University of the Witwatersrand, South Africa; bbbbbbbbb University of the Witwatersrand, South Africa; ccccccccc FIOCRUZ, Brazil; ddddddddd Global Action for Fungal Infections, United Kingdom; eeeeeeeee Unidad Clínica de Enfermedades Infecciosas y Microbiología, Instituto de Biomedicina de Sevilla (IBiS)/CSIC, Hospital Universitario Virgen Macarena; Departamento de Medicina, Universidad de Sevilla, Seville, Spain. CIBERINFEC, Instituto de Salud Carlos III, Madrid, Spain; fffffffff Global Strategy Lab, Canada; ggggggggg Infectious Diseases Unit, 3rd Department Pediatrics, School of Medicine, Faculty of Health Sciences, Aristotle University of Thessaloniki, and Hippokration General Hospital, Thessaloniki, Greece; hhhhhhhhh University of Surrey, United Kingdom; iiiiiiiii Ministry of Health and Wellness, Barbados; jjjjjjjjj IQLS, France; kkkkkkkkk Université de Paris, France; lllllllll University of British Columbia, Canada; mmmmmmmmm Programme for Global Health, Chatham House, London, United Kingdom; nnnnnnnnn Université de Lomé, Togo; ooooooooo Centre d'infectiologie Charles Mérieux, University of Antananarivo, Madagascar; ppppppppp Fondazione Policlinico Universitario A. Gemelli IRCCS, Italy; qqqqqqqqq Department of Surgery, Macerata Hospital, Italy; rrrrrrrrr University of Pretoria, South Africa; sssssssss Radboud University, Netherlands; ttttttttt National Center for Infection Control, Israel Ministry of Health, Israel; uuuuuuuuu Catholic University of Health and Allied Sciences (CUHAS), Mwanza, Tanzania; vvvvvvvvv College of Medicine, Mohammed Bin Rashid University of Medicine and Health Sciences, Dubai, United Arab Emirates; School of Dentistry, Cardiff University, Cardiff, United Kingdom; wwwwwwwww Emory University School of Medicine, United States of America; xxxxxxxxx Aga Khan University, Pakistan; yyyyyyyyy Public Health Agency of Canada, Canada; zzzzzzzzz Chonnam National University Medical School, Republic of Korea; aaaaaaaaaa Research Institute for Tropical Medicine, Philippines; bbbbbbbbbb Centers for Disease Control and Prevention, United States of America; cccccccccc Antimicrobial Pharmacodynamics and Therapeutics, Department of Pharmacology and Therapeutics, University of Liverpool, Liverpool, United Kingdom; Antimicrobial Drug Discovery, Department of Chemistry, The Robert Robinson Laboratories, University of Liverpool, Liverpool, United Kingdom; dddddddddd National Institute of Tuberculosis and Respiratory diseases, New Delhi, India; eeeeeeeeee International Centre for Antimicrobial Resistance Solutions (ICARS); ffffffffff University of Washington, Seattle, United States of America; gggggggggg Faculty of Medicine and University Hospital Cologne, University of Cologne, Germany; hhhhhhhhhh Centers for Disease Control and Prevention, United States of America; iiiiiiiiii Centers for Disease Control and Prevention, United States of America; jjjjjjjjjj UiT The Arctic University of Norway, Norway; kkkkkkkkkk University of Verona, Italy; llllllllll National Tuberculosis Control Program, Pakistan; mmmmmmmmmm International Health Policy Program, Ministry of Public Health, Thailand; nnnnnnnnnn Department of Medical Sciences, Uppsala University, Uppsala, Sweden; oooooooooo University of Melbourne, Australia; pppppppppp University of Oxford, Vietnam; qqqqqqqqqq Ministry of Health, Brazil; rrrrrrrrrr World Health Organization, Geneva, Switzerland; ssssssssss National Health Laboratory Services, Red Cross War Memorial Children's Hospital, Cape Town, South Africa; tttttttttt European University Cyprus, Nicosia, Cyprus; uuuuuuuuuu Aquarius Population Health, United Kingdom; vvvvvvvvvv Cambodia-Oxford Medical Research Unit, Angkor Hospital for Children, Siem Reap, Cambodia; and Centre for Tropical Medicine and Global Health, University of Oxford, Oxford, United Kingdom; wwwwwwwwww National Institute for Communicable Diseases, a division of the National Health Laboratory Service, South Africa; xxxxxxxxxx Erasmus MC, University Medical Center Rotterdam, Department of Medical Microbiology and Infectious Diseases, Netherlands; yyyyyyyyyy National Institute for Public Health and the Environment, Netherlands; zzzzzzzzzz Ha Noi Oxford University Clinical Research Unit, University of Oxford, United Kingdom; aaaaaaaaaaa Christian Medical College, India; bbbbbbbbbbb Radboudumc – CWZ Center of Expertise for Mycology; Center for Infectious Disease Research, Diagnostics and Laboratory Surveillance, the Netherlands; ccccccccccc Department of Parasitology, Universitas Indonesia, Faculty of Medicine, Jakarta, Indonesia; Department of Parasitology, Universitas Kristen Indonesia, Faculty of Medicine, Jakarta, Indonesia; ddddddddddd Peking University People’s Hospital, Beijing, China; eeeeeeeeeee MRC Centre for Medical Mycology, University of Exeter, United Kingdom; fffffffffff No institutional affiliation, United States of America; ggggggggggg East Central and Southern Africa Health Community, Arusha, Tanzania; hhhhhhhhhhh University of Queensland, Australia; iiiiiiiiiii Imperial College London, United Kingdom; United Kingdom Health Security Agency, United Kingdom; jjjjjjjjjjj University of Sydney / Sydney Children's Hospital Network, Australia; kkkkkkkkkkk Zhejiang University, China; lllllllllll Instituto Nacional de Salud, Peru, and Universidad Nacional Mayor de San Marcos, Peru; mmmmmmmmmmm National University of Singapore, Singapore; nnnnnnnnnnn World Health Organization, Geneva, Switzerland; ooooooooooo Sir Run Run Shaw Hospital, School of Medicine, Zhejiang University, China; ppppppppppp National Medicines Institute, Warsaw, Poland; qqqqqqqqqqq World Health Organization, Geneva, Switzerland; rrrrrrrrrrr The University of Edinburgh, United Kingdom; a Antimicrobial Resistance Division, WHO, Geneva, Switzerland; b Global Tuberculosis Programme, WHO, Geneva, Switzerland; c Global HIV, Hepatitis and STI Programme, WHO, Geneva, Switzerland; d Department of Immunization, Vaccines & Biologicals, WHO, Geneva, Switzerland; e Department of Integrated Health Services, WHO, Geneva, Switzerland; f Department of Clinical Research, Faculty of Infectious and Tropical Diseases, London School of Hygiene and Tropical Medicine, London, UK; g Institute of Medical Microbiology, University of Münster, Münster, Germany; h Division of Paediatric Infectious Diseases, Department for Women’s and Children’s Health, University of Padua, Padua, Italy; i Library and Digital Information Networks, WHO, Kobe, Japan; j Public Health Department, Canton of Vaud, Lausanne, Switzerland; k Infectious Diseases Service, Lausanne University Hospital, Lausanne, Switzerland; l Oxford University Clinical Research Unit Indonesia, Faculty of Medicine Universitas Indonesia, Jakarta, Indonesia; m Centre for Tropical Medicine and Global Health, Nuffield Department of Medicine, University of Oxford, Oxford, UK

## Abstract

The WHO research agenda for antimicrobial resistance (AMR) in human health has identified 40 research priorities to be addressed by the year 2030. These priorities focus on bacterial and fungal pathogens of crucial importance in addressing AMR, including drug-resistant pathogens causing tuberculosis. These research priorities encompass the entire people-centred journey, covering prevention, diagnosis, and treatment of antimicrobial-resistant infections, in addition to addressing the overarching knowledge gaps in AMR epidemiology, burden and drivers, policies and regulations, and awareness and education. The research priorities were identified through a multistage process, starting with a comprehensive scoping review of knowledge gaps, with expert inputs gathered through a survey and open call. The priority setting involved a rigorous modified Child Health and Nutrition Research Initiative approach, ensuring global representation and applicability of the findings. The ultimate goal of this research agenda is to encourage research and investment in the generation of evidence to better understand AMR dynamics and facilitate policy translation for reducing the burden and consequences of AMR.

## Introduction

Antimicrobial resistance (AMR) is a global public health hazard with serious economic consequences.^1^^,^^2^ To mitigate the effects of AMR, the WHO member states endorsed a global action plan on AMR in the year 2015, mandating WHO to develop a global public health research agenda for filling major gaps in the knowledge on AMR and monitoring its implementation.^3^

To facilitate evidence generation regarding the pathogens posing the greatest public health threat associated with AMR, WHO published the priority list of bacterial pathogens^4^ in 2017 and of fungal pathogens^5^ in 2022 and updated the priority list of bacterial pathogens^6^ in 2024. In addition, WHO regularly analyses the pipeline for agents that target bacteria and fungi to facilitate priority setting and inform research and development (R&D) efforts.^7^

Despite the increasing focus on AMR research, substantial knowledge gaps continue to hamper an effective response against AMR, as illustrated by the stagnant progress in the implementation of national action plans on AMR,^8^ particularly in low-income and middle-income countries (LMICs), which carry the highest burden of bacterial AMR.^1^^,^^2^ Large data gaps in the prevalence and burden of disease hinder reliable estimates of AMR, particularly in settings with inadequate laboratory capacity and data collection systems.

The pressing need for evidence to inform public health policy, one of WHO’s strategic and operational priorities to address drug-resistant bacterial infections^9^ between the years 2025 and 2035, was recognised by the 77th World Health Assembly in a resolution on accelerating national and global responses to AMR.^10^ Approved on May 30, 2024, the resolution^11^ urges the WHO member states to support and foster basic, applied, and implementation research on infection prevention and control, vaccines, diagnostic tools, therapeutics, and antimicrobial stewardship through collaboration with academia, civil society, and the private sector.

Although research agendas have been successfully developed in other public health areas,^12^, ^13^, ^14^ initiatives aimed at bridging gaps in the research on drug-resistant bacterial infections in human health have fallen short of identifying key research priorities owing to the complex, multifaceted nature of AMR.^15^^,^^16^ Some initiatives focused solely on specific aspects of AMR^15^^,^^17^, ^18^, ^19^, ^20^ or exclusively on the One Health interface,^21^ and priorities specifically relevant to low-resource settings were generally overlooked.

A prioritised, multidisciplinary research agenda on AMR is warranted to direct investments of governments and other research funders and to channel the attention of policy makers, researchers, and the private sector towards crucial knowledge gaps that demand evidence generation, which, in turn, could inform the development and effective implementation of AMR policies and interventions. We hereby present the first comprehensive effort focusing on infections caused by drug-resistant bacteria and fungi, developed with a robust and validated methodology for research prioritisation.

## The WHO research agenda for AMR in human health

The research agenda aims to identify the most pressing global research priorities directly related to human health and with the greatest potential to influence policies, interventions, and tools to address AMR in fungal and bacterial priority pathogens identified by WHO, including drug-resistant *Mycobacterium tuberculosis*^22^ and *Mycoplasma genitalium* (panel 1).

Panel 1 **Research agenda development framework**

**Objectives**

The research agenda has two objectives: to advance research on antimicrobial resistance (AMR) through identification and prioritisation of research topics encompassing the prevention, diagnosis, treatment and care, and burden and drivers of AMR and optimising intervention delivery; and to encourage increased investment and scientific interest among researchers, donors, and public health professionals.

**Scope and focus**

The research agenda is global in its scope and focuses on AMR in the human health sector, specifically infections caused by WHO bacterial and fungal priority pathogens of crucial importance for AMR and drug-resistant *Mycobacterium tuberculosis* and *Mycoplasma genitalium*. Other pathogens such as drug-resistant viruses and parasites are beyond the scope of the framework.

**Population**

The research agenda aims to define priorities for research applicable to the general population, including neonates and children.

**Geographical context**

Although global in scope, the research agenda places a particular focus on identifying knowledge gaps and prioritising research topics relevant for low-resource settings.

**Timeframe**

The research agenda aims to align its timeline with the Sustainable Development Goals, recognising the 2030 deadline for achieving both these global targets, and to address prioritised research topics.

**Research investment strategy**

The research agenda aims for investments in AMR research to be balanced and diversified across the prioritised research topics (ie, not focusing on one or a few high-risk or expensive research ideas or both) and encompassing the four domains of research (ie, descriptive, delivery, discovery, and development).

**Figure 1:**
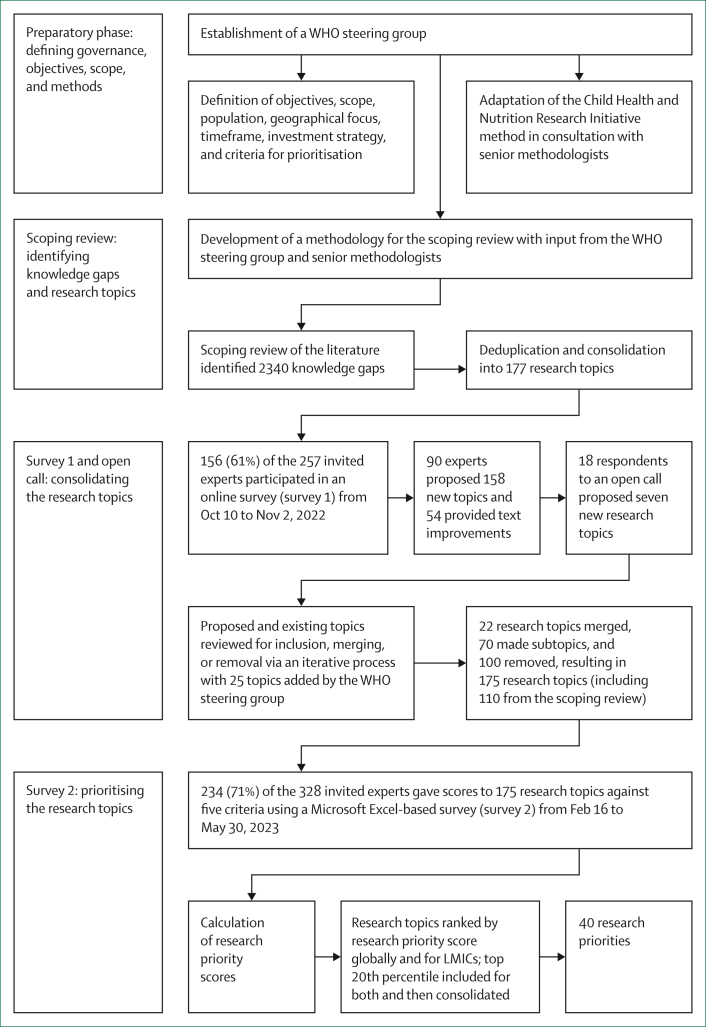
Identification of the 40 research priorities in the WHO agenda for research on antimicrobial resistance and human health LMICs=low-income and middle-income countries.

The research agenda places particular emphasis on bridging the crucial knowledge gaps by the year 2030, in alignment with the global Sustainable Development Goals, and on research relevant for low-resource settings, in recognition of the disparities in accessibility, availability, and utilisation of diagnostics, antimicrobials, and measures for prevention of infection. The research agenda encompasses four research domains—description, delivery, development, and discovery research^12^^,^^13^^,^^23^—and 11 AMR areas to comprehensively address the multifaceted challenges posed by AMR to human health. The development process of the research agenda (figure 1) encompasses four steps: preparatory phase, scoping review, consolidation of research topics (ie, survey 1), and research prioritisation (ie, survey 2).

### Preparatory phase: defining governance, objectives, scope, methods, and establishment of the expert group

A multidisciplinary WHO steering group was established to oversee the entire development process, including defining the objectives, scope, population, geographical focus, timeframe, AMR areas, and prioritisation criteria.

We adopted the widely used priority-setting method known as the Child Health and Nutrition Research Initiative (CHNRI).^12^^,^^13^^,^^23^ This expert survey-based scoring system offers a systematic, transparent, and reproducible approach for prioritising research topics from a large pool of topics by a large group of experts. CHNRI offers an advantage over consensus-based approaches by mitigating individual opinion bias through independent scoring (panel 2).^23^^,^^24^

Panel 2 **Approaches for agenda priority setting**

**Consensus-based approaches**

Consensus-based approaches lead to priorities decided by means of group consensus.

Strengths: Priorities decided by means of group consensus, which improves acceptability

Weaknesses: Not strictly systematic; might result in obvious priorities without strong evidence; priorities might reflect the views and biases of only a few experts and, potentially, those who are more vocal in the discussions; not feasible for prioritisation of a large number of research topics

Examples: Combined approach matrix and priority-setting partnerships

Usage: More often used for national prioritisation exercises

**Metric-based approaches**

Metric-based approaches generate priorities on the basis of an algorithm that pools individual scoring of research questions.

Strengths: Systematic and repeatable; reduces the risk of an individual opinion dominating over others; more feasible for a larger number of stakeholders to participate and dampens down the dominance of a minority but vocal stakeholders

Weaknesses: Scoring can be demanding for participants; individuals can score in isolation and can limit opportunities for dialogue

Examples: Child Health and Nutrition Research Initiative and Delphi techniques

Usage: More suitable for global prioritisation exercises

**Figure 2:**
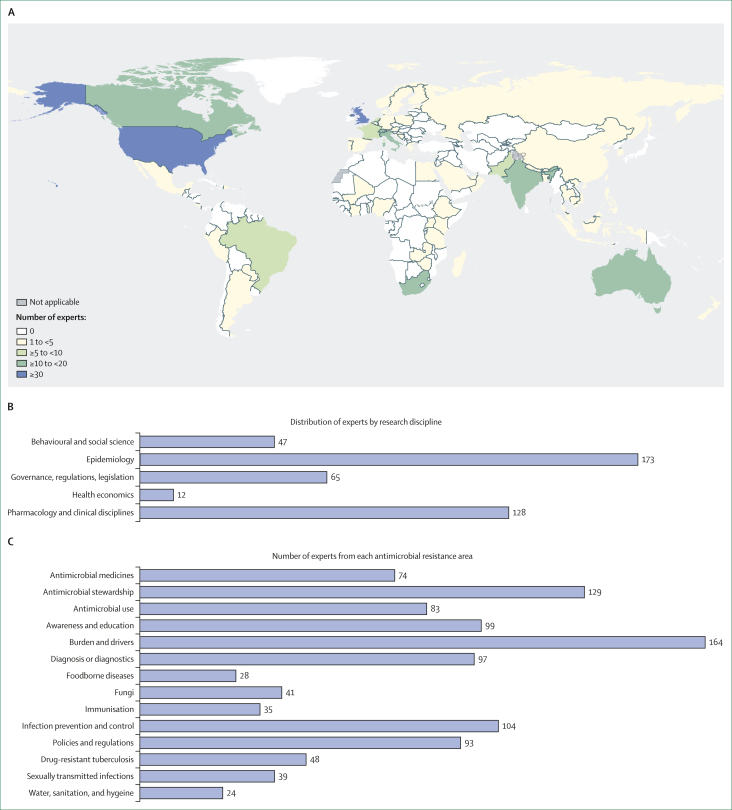
Details of experts involved (A) Geographical distribution of experts. (B) Number of experts by research discipline (data missing for 15 experts). (C) Number of experts in each antimicrobial resistance area (experts could report more than one research discipline and area of expertise).

A group of global experts was established through a combination of systematic Web of Science searches and referrals (appendix pp 2–5), seeking a diverse range of expertise across research domains, AMR areas, geographical representation, income settings, and populations. Among the 560 experts identified, 261 (47%) contributed to the development of the research agenda (survey 1, survey 2, or both). Experts were from 69 countries across all WHO regions—European region (41%), region of the Americas (24%), African region (15%), Western Pacific region (8%), South-East Asia region (7%), and Eastern Mediterranean region (4%)—with the highest representation from the UK, USA, South Africa, Switzerland, Canada, India, Brazil, and Australia (figure 2A).

### Identifying knowledge gaps and research topics: a scoping review

The initial CHNRI step entailed experts submitting research proposals or ideas. We augmented this step with a comprehensive scoping review of peer-reviewed and grey literature published in the preceding 10 years (2012–21) and included a comprehensive search for systematic and scoping reviews indexed in PubMed, Embase, and Web of Science, as well as manual searches of data repositories and websites of 92 key organisations. The detailed findings of the scoping review have been presented elsewhere.^25^^,^^26^ In brief, the review identified 2340 knowledge gaps (panel 3) sourced from a pool of over 3000 documents. The knowledge gaps covered the burden, drivers, technologies, tools, and interventions for the prevention, diagnosis, and treatment of infections caused by drug-resistant pathogens, as well as care of affected individuals within the scope of this research agenda. These knowledge gaps were subsequently categorised into research topics or subtopics, depending on the level of granularity offered. The topics were further arranged into the population, intervention or exposure, comparator, outcome (PICO/PECO) format and consolidated into 177 thematic research topics.^25^^,^^26^

Panel 3 **Definitions**

**Knowledge gap**

Knowledge gaps refer to areas with incomplete understanding, information, or data. Identifying research gaps helps to formulate relevant and pressing research questions.

**Research topic**

A research topic encompasses multiple research questions. Rather than focusing on a single inquiry, a research topic involves exploring various facets or aspects of a broader research area.

**Research priority**

Research priorities are specific research topics that are deemed particularly important or urgent to address. These priorities guide researchers, policy makers, and funding agencies in allocating resources and efforts to areas that would benefit the most from additional evidence or that contain the most substantial knowledge gaps.

**Research domain**

Four research domains often used by the Child Health and Nutrition Research Initiative knowledge matrix to categorise research topics:

• Descriptive (have greater understanding of antimicrobial resistance [AMR] burden and drivers)
• Delivery (provide ways to deliver existing interventions with better quality)
• Development (improve existing interventions, reduce their costs, or optimise their implementation)
• Discovery (identify new interventions to prevent, treat, and diagnose AMR)

**AMR theme**

The research topics and priorities cover three themes—AMR prevention, diagnosis, and treatment and care—in accordance with the WHO people-centred approach for addressing AMR, in addition to an overarching theme covering AMR epidemiology, burden and drivers, awareness and education, and policies and regulations.

**AMR area**

The research topics and priorities encompass areas relevant to AMR: water, sanitation, and hygiene; infection prevention and control; immunisation; diagnosis or diagnostics; antimicrobial stewardship; antimicrobial use; antimicrobial medicines; epidemiology, burden, and drivers of AMR; awareness and education; policies and regulations; and prevention, diagnosis, and treatment of drug-resistant tuberculosis, as well as care of individuals with drug-resistant tuberculosis.

**WHO steering group**

A group of WHO staff with technical expertise across various AMR areas. This group defined the objectives and scope of the agenda, supported the consolidation of research topics, developed the criteria for prioritisation, and guided the prioritisation process.

**Expert group**

A group of global experts external to WHO identified by means of a systematic search of publication output in Web of Science and referrals, with a mix of expertise from across research disciplines and AMR areas, geographical representation, income settings, and paediatric expertise. The expert group participated in the identification of knowledge gaps (survey 1) and prioritisation of research topics (survey 2).

The research topics were then organised into a knowledge matrix to categorise each topic into the four CHNRI-defined research domains (descriptive, delivery, development, and discovery research)^12^^,^^13^ and three themes (prevention, diagnosis, and treatment and care) according to the WHO people-centred approach for AMR.^27^

### Consolidating the research topics: survey 1 and open consultation

Through an online survey (survey 1) conducted from Oct 10 to Nov 2, 2022, 156 experts provided their inputs on the 177 research topics identified through the scoping review (appendix p 5). For each topic, experts could choose to agree with its inclusion, suggest text improvements, or recommend removal under the conditions that the topic was deemed to have sufficient existing evidence, be unlikely to inform policies or interventions, or be unfeasible to design and execute a research study and generate evidence^27^ by the year 2030. In addition, expert group members could suggest up to two additional topics meeting five criteria—ie, a new topic not covered already in the list; addressing a crucial knowledge gap; relevant to human health; relevant for informing policy; and feasible to generate evidence by the year 2030.

Each research topic in survey 1 received recommendations for removal from at least one expert, with a median of seven suggestions (range 1–34) per topic. Additionally, 158 new topics were proposed by 90 experts, and 54 experts offered suggestions for improvement in existing topics. In parallel, we launched an open call for expert contributions via the WHO website to engage the broader research community.^28^ The open call enabled individuals to review existing topics, propose new ones, and evaluate the proposed topics on the basis of the specified criteria. This process led to 18 individuals suggesting the removal of 58 topics and proposing seven new topics.

In addition to the 177 research topics identified from the scoping review, proposals from 165 new topics came from both the expert survey and open call. The topics underwent a rigorous iterative review on the basis of a semistructured decision-making process, involving subject area experts in the WHO steering group to agree on acceptance or rejection. This process resulted in 110 scoping review topics and 65 new topics being retained, 100 topics (from the scoping review, survey, or open call) being removed, and 70 topics being reclassified as subtopics. 22 topics of similar nature (albeit providing a slightly different perspective on the same subject) were combined with existing topics into composite research topics, which would require various types of studies to address them effectively. The steering group evaluated the revised list and suggested an additional 25 topics. Discrepancies among the topics were resolved through face-to-face or online meetings or via email, resulting in a final consolidated list of 175 topics.

### Prioritising the research topics: survey 2

In survey 2, conducted from Feb 16 to May 30, 2023, 234 experts assigned scores for the 175 consolidated research topics (appendix p 5). Notably, 129 of these experts had also participated in survey 1. The experts specified their primary perspective (high-income, low-income and middle-income, or all income settings) and age focus (adults, children, or all age groups). In scoring the topics, 93 (40%) of 234 applied an LMIC perspective, 86 (37%) considered all income settings, and the remaining 55 (23%) applied a high-income settings perspective. Additionally, 78 (33%) experts focused on adults only, 38 (16%) focused on neonates or children only, and 118 (50%) considered all age groups in their scoring. Among the 234 experts participating in survey 2, 114 (49%) identified as women and 84 (36%) were affiliated with institutions in LMICs. The number of experts by research disciplines and AMR expertise are shown in figure 2B, C.

The experts assessed each individual research topic against five criteria (panel 4) and assigned their responses as: yes (score, 1 point), no (score, 0 point), or do not know. Intermediate criterion scores for each topic were calculated by summing the scores for each criterion and dividing by the number of experts who scored that topic and criterion, excluding blank or do-not-know responses.^12^^,^^13^ We repeated this calculation with input exclusively from experts assigning scores on topics from the perspective of LMICs. We then calculated a research priority score (RPS) for each topic from both global and LMIC perspectives. The RPS was calculated as a weighted average of the five criterion scores, with feasibility by 2030 and health equity carrying a weight of 10% each and the remaining three criteria carrying a weight of 27% each. This weightage distribution, following the methodology described by Rudan and colleagues,^13^ acknowledges some heterogeneity in the scoring. Notably, the interpretation of criterion feasibility by the year 2030 might not be consistent among experts, as research endpoints could be interpreted as study initiation, completion, or publication. Similarly, the health equity criterion might not be uniformly applied and could be perceived to be too abstract and difficult to measure. The topics were ranked into two separate lists on the basis of the weighted RPS: one global (irrespective of income setting) and one considering responses solely from experts assessing topics from an LMIC setting perspective.

Panel 4 **Criteria for scoring and prioritising research topics**

**Filling** **crucial** **knowledge gaps**

Is the research most likely to fill crucial knowledge gaps on antimicrobial resistance (AMR)?

**Answerability and feasibility by the year 2030**

Can a research study be designed and carried out by the year 2030 to address at least some components of the research topic? (Funding considerations should be disregarded.)

**Potential for translation into policy**

Do the findings have the potential to inform evidence-based policy and practices aimed at mitigating the effects of AMR on public health?

**Effect on mitigating AMR**

Is the research most likely to result in an intervention that is effective, efficacious, or efficient or combinations of these in mitigating the effects of AMR? (Effective: producing a desired effect in a real-world environment; efficacious: providing a desired effect in a controlled environment; efficient: providing highest value for the available resources.)

**Promoting health equity**

Is the research most likely to promote health equity (ie, leading to interventions that will reduce the inequitable effects of AMR across diverse socioeconomic contexts and underprivileged populations)?

Overall, the median number of experts scoring each topic (across all five criteria) was 140 (range 102–183). Across all the 175 research topics, the median RPS was 0·85 (0·62–0·95), and 130 (74%) topics had an RPS of 0·80 or higher. For LMICs, the median RPS was 0·90 (0·70–0·97), with 162 (92%) topics having an RPS of 0·80 or higher. Following the ranking of topics based on descending RPS, the highest-scoring topics within the top 20th percentile were evaluated for potential inclusion in the definitive priority list. This threshold was selected to ensure a manageable number of research priorities with representation across all AMR areas and resulted in 35 globally ranked topics and an additional list of 35 topics from an LMIC settings perspective. We merged duplicates among the 70 identified topics, resulting in 40 research priorities for inclusion in the research agenda, addressing both global and LMIC perspectives. The prioritisation results were shared with the WHO steering group and the expert group via email and presented at a WHO global webinar.

## Overview of the research priorities

The 40 research priorities include seven priority topics focused on drug-resistant tuberculosis and 33 on drug-resistant bacterial and fungal infections. The bacterial and fungal priorities are divided into prevention (n=4), diagnosis (n=6), treatment and care (n=11), and overarching themes, including AMR epidemiology, burden, and drivers, awareness and education, and policies and regulations (n=12; panel 5).

Panel 5 **40 research priorities for antimicrobial resistance in human health across five themes and 11 areas, with research priority scores [RPSs] (the order in which research priorities are presented is unranked)**

**Prevention**

*Water, sanitation, and hygiene (WASH)*

Investigate the effect, contribution, utility, effectiveness, and cost-effectiveness of interventions to ensure safe WASH (including hand hygiene) and waste management practices in the community setting on reducing the burden and drivers of antimicrobial resistance (AMR), such as unnecessary antibiotic use for diarrhoeal diseases in low-income and middle-income countries (LMICs; RPS=0·90∗)

Investigate implementation strategies of WASH-related interventions in health-care settings (including ensuring access to safely managed water and sanitation, safe hand hygiene, and safe management of waste and environmental cleaning), and assess their effects, acceptability, equity, and cost-effectiveness on the burden and transmission of antimicrobial-resistant infections associated with health-care settings and prescription of antimicrobial drugs across socioeconomic settings (RPS=0·89∗)

*Infection prevention and control*

Identify the most effective, cost-effective, acceptable, and feasible multimodal infection and prevention control strategies (such as hand hygiene, contact precautions, and patient isolation) and the relative effect of their components in reducing different types of infections associated with health-care settings caused by multidrug-resistant pathogens across geographical and socioeconomic settings (RPS=0·90∗)

*Immunisation*

Assess the effect of vaccines on preventing colonisation and infection by drug-resistant pathogens (whether specifically targeted by the vaccine or not) and on reducing the overall use of antimicrobial drugs, and health-care encounters and health system costs among adults and children and across socioeconomic settings (RPS=0·94†)

**Diagnosis**

*Diagnosis or diagnostics*

Investigate and evaluate rapid point-of-care diagnostic tests (including biomarker-based tests) and diagnostic algorithms to distinguish between bacterial and viral infections and non-infectious syndromes that are feasible for use in low-resource settings and among different subpopulations (including children and neonates) and their effects on clinical outcomes (RPS=0·89∗)

Investigate and evaluate diagnostic tests for isolating, identifying, testing the antimicrobial susceptibility, or detecting the resistance of bacterial pathogens, or any combination of these, including multiplex panel-based tests and tests using novel technologies that are fast, (near) point-of-care, affordable, feasible for use in low-resource settings and among different subpopulations and for various specimen types, and their effects on clinical outcomes (RPS=0·91∗)

Investigate and evaluate phenotypic and genotypic methods of rapid antimicrobial susceptibility testing and resistance detection directly from positive blood culture bottles, especially for use in LMICs and their effects on clinical outcomes (RPS=0·89∗)

Investigate and evaluate rapid, (near) point-of-care diagnostic tests (including antigen-based and multiplex panel-based tests) for detecting WHO fungal priority pathogens^5^ of crucial importance for AMR (such as *Candida auris*, *Aspergillus fumigatus*, and *Cryptococcus neoformans*) that are feasible for use in low-resource settings and among different subpopulations and their effects on clinical outcomes (RPS=0·89∗)

Investigate and evaluate the clinical utility and diagnostic accuracy of phenotypic antifungal susceptibility testing (including identifying minimal inhibitory concentration breakpoints and testing for in-vitro synergy and in-vivo synergy between antifungal drugs) and its effects on clinical outcomes (RPS=0·94†)

Investigate, assess the performance, and evaluate the implementation of novel rapid point-of-care molecular and non-molecular assays, and optimal testing and screening approaches (including self-testing) for *Neisseria gonorrhoeae* and AMR detection, to reduce inappropriate prescription of antibiotics and emergence of AMR (RPS=0·90∗)

**Treatment and care**

*Antimicrobial stewardship*

Investigate context-specific, feasible, sustainable, effective, and cost-effective antimicrobial stewardship interventions (such as implementing WHO access, watch and reserve [AWaRe] antibiotic book,^27^ guidelines, clinical algorithms, education and training, audit, and feedback), alone or in combination, to avoid antimicrobial misuse in outpatient and inpatient settings, especially under situations with inadequate diagnostic capacity (RPS=0·95∗)

Identify feasible, effective, and scalable pharmacist antimicrobial drugs-dispensing practices and related regulatory frameworks to improve antimicrobial stewardship in the community, especially in LMICs (RPS=0·90∗)

Investigate criteria and strategies to optimise empirical antimicrobial therapy for important infectious syndromes, such as bloodstream, upper and lower respiratory tract, urinary tract, skin and soft tissue, CNS, and sexually transmitted infections, especially in settings with inadequate medicine availability, diagnostic capacity, and access to health-care services (RPS=0·92∗)

*Antimicrobial use*‡

Identify optimal (feasible, accurate, and cost-effective) methods and metrics to monitor antimicrobial use in the community and health-care settings and appropriate targets to monitor progress in reducing inappropriate antimicrobial use (RPS=0·88∗)

Determine the levels, patterns, trends, and drivers of appropriate and inappropriate prescribing of AWaRe antibiotics^29^ across countries and community and health-care settings, with data disaggregated on the basis of sex, age, socioeconomic status and subpopulations, including those that are experiencing vulnerability§ (RPS=0·91∗)

Investigate optimal approaches to effectively use facility-level, national-level, or their combination antimicrobial use and resistance surveillance data to inform antimicrobial stewardship programmes and treatment guidelines (RPS=0·91∗)

*Antimicrobial medicines*

Investigate efficacious and safe antibiotic treatment regimens for infections on the basis of old and new agents (and their combinations), especially those of extended-spectrum β-lactamase-producing or carbapenem-resistant Enterobacterales, or both, with minimum selection and transmission risk for AMR, especially among children and other clinically vulnerable subpopulations (RPS=0·90∗)

Investigate efficacious and safe antibiotic treatment regimens for infections by drug-resistant typhoid and non-typhoidal salmonella (including pathogens resistant to cephalosporins and fluoroquinolones) across socioeconomic settings (RPS=0·93†)

Investigate efficacious and safe empirical antibiotic treatment (drug choice, drug combination, route, dose, and duration) for Gram-negative bacteria causing bloodstream infections or sepsis among neonates and young children, especially in settings with high AMR prevalence and inadequate diagnostic capacity and antimicrobial drug availability (RPS=0·90∗)

Investigate antifungal regimens optimised for efficacy, cost, safety, and duration for the treatment of infections caused by WHO fungal priority pathogens^5^ of crucial importance for AMR (such as *C auris*, *A fumigatus*, and *C neoformans*) in settings with increasing or high prevalence of antifungal resistance (RPS=0·89∗)

Investigate efficacious and safe regimens on the basis of new or existing antimicrobial medicines for urogenital and extragenital sexually transmitted infections (such as drug-resistant *N gonorrhoeae* or *Mycoplasma genitalium*) in the context of increasing AMR levels, including in populations experiencing vulnerability§ (RPS=0·89∗)

**Overarching topics**

*AMR epidemiology, burden, and drivers*

Investigate the prevalence, incidence, mortality, morbidity, and socioeconomic effects of community-acquired infections (respiratory tract, urinary tract, and bloodstream infections) and infections associated with health-care settings (bloodstream, urinary tract, surgical site, and respiratory tract infections) by resistant WHO bacterial priority pathogens (*Acinetobacter baumannii*, *Pseudomonas aeruginosa*, Enterobacterales, *Enterococcus faecium*, *Staphylococcus aureus*, *Helicobacter pylori*, *Campylobacter* spp, *Salmonella* spp, *N gonorrhoeae*, *Streptococcus pneumoniae*, *Haemophilus influenzae*, and *Shigella* spp),^4^ with data disaggregated on the basis of sex, age, socioeconomic status, and in subpopulations experiencing vulnerability§ and across socioeconomic settings, especially in LMICs (RPS=0·91∗)

Investigate the prevalence, incidence, morbidity, mortality, and socioeconomic effects, and identify and quantify the routes and dynamics of infections by drug-resistant WHO fungal priority pathogens^5^ of crucial importance for AMR (such as *C auris*, *A fumigatus*, and *C neoformans*), across geographical and socioeconomic settings and in populations experiencing vulnerability§ (RPS=0·94†)

Investigate the association, contribution, and effects of structural and health system factors (such as hospital microbiome, sanitation infrastructure, waste management, health expenditure, governance, distribution of resources, population displacement, conflict, and disruptions in the care continuum) on colonisation (selection, persistence, and spread or loss of bacterial populations) and infection by WHO bacterial^4^ and fungal^5^ priority pathogens in various subpopulations, including those experiencing vulnerability§ and people with comorbidities, across various socioeconomic settings (RPS=0·94∗)

Identify optimal (efficient, effective, and cost-effective) surveillance methods to generate accurate and reliable data on the epidemiology and burden of AMR among WHO bacterial^4^ and fungal^5^ priority pathogens (including identifying the genotypic predictors of resistance) in community and health-care settings and disaggregated on the basis of sex, age, and subpopulations; moreover, these data should be relevant and actionable at the local and national levels, especially in LMICs (RPS=0·94∗)

Assess the short-term and long-term effects of the programmatic use of antimicrobial drugs in mass administration on AMR, focusing on clinically susceptible subpopulations in low-income settings (RPS=0·90∗)

Evaluate the public health benefits, cost, and effects on unnecessary or inappropriate prescription of antibiotics, and potential AMR consequences of currently recommended syndromic sexually transmitted infection management and treatment of individuals with asymptomatic sexually transmitted infections (including *N gonorrhoeae* infection) in settings with variable diagnostic capacity (RPS=0·95†)

*AMR awareness and education*

Identify the most cost-effective behavioural change interventions to mitigate the emergence and spread of AMR by targeting and engaging the general public, young people, mass media, health-care providers, and policy makers across socioeconomic settings (RPS=0·94∗)

*Policies and regulations related to AMR*

Evaluate the implementation of AMR-related policies and regulations at the national level and their effectiveness in mitigating AMR and improving health outcomes in the community and health-care settings across socioeconomic settings (RPS=0·93†)

Investigate strategies for the sustainable and cost-effective implementation of national policies, legislation, and regulations (including sustainable financing and optimal governance structures) to improve infection prevention and patient care practices and the use of antimicrobial drugs in the community and health-care settings, across socioeconomic settings (RPS=0·89∗)

Identify the most cost-effective interventions to mitigate AMR in the human health sector, globally and within countries or regions, and identify the rationale, costs, benefits, feasibility, sustainability, and potential returns on investment to achieve the greatest benefit (RPS=0·89∗)

Investigate strategies to integrate AMR interventions into broader health, health financing, development, welfare structures and national policies, and evaluate their effects on mitigating AMR, enhancing health system efficiency, reducing people’s out-of-pocket expenses, and improving equitable access to and use of diagnostics and antimicrobial drugs (RPS=0·96†)

Investigate how existing regulatory frameworks, marketing incentives (or their absence), and sustainable financing models affect the development and availability of new antimicrobial drugs and identify effective strategies to adapt these approaches to low-income settings to improve their availability for adults and children (RPS=0·96†)

**Drug-resistant tuberculosis**

*Prevention*

Investigate effective preventive tuberculosis vaccines that meet the criteria of WHO-preferred product characteristics and show effects on preventing infection, disease, and recurrence (relapse or reinfection), thereby preventing or reducing the incidence of drug-resistant tuberculosis (RPS=0·89∗)

*Diagnosis*

Investigate how the diagnostic performance of molecular assays can be improved to detect drug resistance among individuals with extrapulmonary and pulmonary tuberculosis caused by non-respiratory specimens, including among children and adolescents (RPS=0·94†)

Identify optimal diagnostic and treatment delivery models to improve the access, effectiveness, cost-effectiveness, feasibility, and acceptability of drug-resistant tuberculosis testing and treatment across settings and subpopulations experiencing vulnerability§ (such as people living with HIV, children and adolescents, and prisoners) and evaluate their effects on reducing drug-resistant tuberculosis at the population level (RPS=0·90∗)

*Treatment and care*

Investigate better tolerated, optimally dosed, more effective, and shorter combination regimens using a stratified risk approach for treating all forms of drug-resistant tuberculosis, including in socioeconomically or clinically vulnerable populations (RPS=0·92∗)

Identify the optimal, cost-effective, shortest duration, and safest tuberculosis-preventive treatment for the contacts of individuals with drug-resistant tuberculosis, especially among individuals at high risk of tuberculosis infection and disease, as identified in WHO guidance, and eligible clinically vulnerable or susceptible populations (such as children, adolescents, people living with HIV, and pregnant women; RPS=0·91∗)

Investigate strategies for improving treatment outcomes among people with drug-resistant tuberculosis who have known risk factors and co-occurring conditions (such as HIV, undernutrition, diabetes, tobacco use, alcohol and other substance use, and mental health disorders) and clinically susceptible populations (such as pregnant and breastfeeding women, children and adolescents, and prisoners) in various geographical and socioeconomic settings (RPS=0·97†)

Investigate the programmatic effectiveness, safety, and tolerability of currently used WHO-recommended treatment regimens for drug-resistant tuberculosis (including combinations that contain bedaquiline and pretomanid or delamanid) on patient outcomes and the emergence of drug-resistant tuberculosis across populations and settings and identify the drivers of treatment failure (RPS=0·95†)

∗RPS was within the top 20th percentile upon considering the responses of all experts. †RPS was within the top 20th percentile upon considering the responses of experts giving scores with a low-income and middle-income setting perspective only. ‡Antimicrobial use refers to antimicrobial use data (elsewhere referred as antimicrobial consumption data) that provide information on the quantity and types of antimicrobials used in the settings in which they are administered as well as clinical data linking antimicrobial use patterns, regimens, dose, route, duration, and indication with patient clinical characteristics. §Populations that are experiencing socioeconomical and clinical vulnerability include children, pregnant women, breastfeeding mothers, people living in remote or rural settings, migrants, refugees, prisoners, and people experiencing tuberculosis or malaria and people living with HIV. Relevant populations might differ by topic.

### Prevention

Under the theme of prevention, two research priorities on water, sanitation, and hygiene (WASH) address the need to investigate the implementation and effect of WASH-related interventions on the burden of AMR and antimicrobial prescription in community and health-care settings. The infection prevention and control priority focuses on identifying multimodal intervention strategies and evaluating the relative effect of their components. The research priority on immunisation highlights the need to evaluate the effects of vaccines on colonisation, infection with resistant organisms, and their indirect effects in strengthening stewardship efforts and reducing the prescription of antimicrobials.

### Diagnosis

Under the theme of diagnosis, six priorities focus on better, accessible, and affordable diagnostics, including point-of-care tests for distinguishing bacterial and viral infections and rapid phenotypic and molecular methods for bacterial identification and antimicrobial susceptibility testing. These topics reflect the stagnant progress in effective diagnosis, with nearly half of the world’s population living with little or no access to diagnostics.^30^ Two priorities focus on the need for better detection and susceptibility testing of WHO fungal priority pathogens of crucial importance for AMR.^31^ One priority addresses the need to better understand the performance of and approaches to implement point-of-care tests for *Neisseria gonorrhoeae.* These tests should contribute to improved identification and management of individuals with potentially asymptomatic sexually transmitted infections, thus reducing transmission and unnecessary use of antibiotics and helping to identify the emergence and transmission of AMR.

### Treatment and care

Under the theme of treatment, three priorities pertain to antimicrobial stewardship target interventions to minimise antimicrobial misuse and strategies to optimise empirical treatment. This emphasis is particularly important in settings with inadequate diagnostic capacity and access to health-care services. The research agenda emphasises the need for studies on pharmacy-level interventions and higher-level frameworks regulating antimicrobial dispensing to improve antimicrobial stewardship in primary care. Three priorities address the need to identify optimal methods and metrics for monitoring antimicrobial use and approaches to effectively use facility-level and national-level surveillance data on antimicrobial use to inform treatment guidelines and stewardship programmes. Five research priorities encompass the investigation of new and existing antimicrobials, with special emphasis on enhancing the treatment of drug-resistant Enterobacterales, including typhoidal and non-typhoidal salmonella. Furthermore, the research priorities focus on infections caused by sexually transmitted and fungal priority pathogens as well as bloodstream infections caused by Gram-negative bacteria in neonates and young children.^32^

### Overarching topics

The availability of reliable AMR surveillance data, and therefore the understanding of AMR, can vary markedly between high-resource and low-resource settings.^1^ Six research priorities focus on understanding the epidemiology, burden, and drivers of drug-resistant bacterial and fungal pathogens and identifying optimal AMR surveillance methods for obtaining accurate and representative data. These research priorities are central for developing evidence-based treatment guidelines and measuring the success of interventions aimed at mitigating the emergence of drug resistance and transmission. Furthermore, the effect of addressing these research priorities on antimicrobial prescription and AMR needs to be investigated. An additional priority raises issues on the effects of programmatic use of antimicrobials in mass or targeted administration on the development of drug resistance and its persistence. The agenda recognises the importance of behavioural change interventions in mitigating AMR and identifying the interventions that might improve AMR awareness and education among both the public (including young people and through mass media) and health-care providers.

Five research priorities address policies and regulations related to AMR. Although studies have assessed the gaps and opportunities to strengthen national-level governance efforts and AMR national action plans,^33^^,^^34^ the agenda emphasises the need for context-specific evidence for formulation of effective strategies to implement national AMR policies and programmes and facilitate a more customised approach to policy implementation. The actions in low-resource settings are often based on evidence from high-income countries; however, knowledge gaps are especially evident in low-resource settings since the availability of resources, socioeconomic and political context, and cultural drivers of antimicrobial use differ between the two settings. Furthermore, information on cost-saving or cost-effective AMR interventions in both hospital and community settings would inform rational policy design. Although evidence from modelling studies in high-income settings exists, data from real-world observational studies and low-resource settings are scarce.^35^, ^36^, ^37^ Evidence-based strategies to effectively integrate AMR interventions into existing national health system structures, thereby avoiding a siloed AMR programme, are warranted to foster financial sustainability of the AMR response.

An important research priority is related to evaluation of the effect of regulatory frameworks, marketing incentives (including pull incentives), and sustainable financing models on antimicrobial R&D pipelines, considering that push incentives alone are deemed insufficient to ensure a healthy pipeline.^38^^,^^39^ Although countries such as Sweden and the UK have been experimenting with novel economic incentives,^40^, ^41^, ^42^ ensuring equitable access to antimicrobials globally would require research into mechanisms that are feasible even in low-resource settings.

Recognising the crucial role of R&D in the areas of vaccination, diagnosis, and treatment, the agenda calls for enhanced efforts to develop new agents against extended-spectrum β-lactamase-producing or carbapenem-resistant Enterobacterales or both, as well as against sexually transmitted infections. The agenda emphasises the need to continue investing in product-related R&D and to expand funding support across the entire research spectrum, from descriptive studies to delivery, development, and discovery.

## Drug-resistant tuberculosis

Seven research priorities address the need for improved tools to prevent, diagnose, and treat drug-resistant tuberculosis. These priorities are consistent with the evidence gaps identified in the consolidated guidelines on tuberculosis put forth by the Guidelines Development Groups of WHO^43^^,^^44^ and relate to the discovery and development of effective vaccines; more effective, safer, and shorter regimens to treat all forms of drug-resistant tuberculosis; prevention of infection among clinically susceptible individuals; and development of point-of-care tests to detect tuberculosis.

## Implications of the research agenda for policy

This paper describes the first WHO research agenda setting global research priorities for AMR in human health, relevant to drug-resistant bacterial and fungal infections of crucial importance for AMR, including drug-resistant *M tuberculosis.* These research priorities encompass the complete people-centred journey, from prevention to diagnosis and treatment, and address overarching knowledge gaps in AMR epidemiology, burden and drivers, policies and regulations, and awareness and education, with a focus on low-resource settings and socioeconomically or clinically vulnerable populations.

The agenda is expected to guide policy makers, the global research community, industry, and research funders in converging research efforts and investments on the most crucial global knowledge gaps. An essential criterion for prioritisation was the potential for translation of research outputs into policy, such that a pronounced effect on AMR prevention practices; the diagnosis, treatment, and management of infections; and national and global policies aiming to mitigate AMR is seen on addressing the research priorities.

The agenda will be instrumental in advancing the discussions and initiatives related to AMR at the UN General Assembly, ensuring that crucial knowledge gaps are addressed by the year 2030 and contributing to national and global strategies for AMR containment and management. Furthermore, evidence^45^ from well designed, large-scale, multidisciplinary studies on the drug-resistant tuberculosis research priorities will support the achievement of the ambitious declarations of the WHO member states to expand coverage of rapid diagnostic testing, treatment, and tuberculosis-preventive treatment to reach 90% and coverage of health and social benefits packages for people with tuberculosis to reach 100% by the year 2027.

Investments in AMR research to date have been inconsistent.^38^ Between January, 2017, and September, 2021, the Global AMR R&D Hub reported a total investment of US$ 8·9 billion across 12 093 projects in the field of AMR by 214 organisations worldwide.^46^ Approximately one-third of the investment in priority bacterial pathogens (equivalent to $4·3 billion) was allocated towards development of novel therapeutics. By contrast, only $530 million (12%) was designated for vaccines, $261 million (6%) for diagnostics, and $32 million (1%) for policy research. In addition, research on fungal infections across all sectors attracted only $512 million (6%) of all investments. Although still lower than that for other disease areas, $8·1 billion (90%) of the investments are directed towards R&D targeting bacterial pathogens, with the top research areas in terms of funding volume across all sectors being basic research and therapeutics R&D.^46^ Investment in product-related R&D such as diagnostics and vaccines is lagging.

Concerted efforts to implement this research agenda have the potential to ensure a more balanced approach to AMR research funding—ie, one that is focused on developing not only an arsenal of therapeutic options but also tools to promptly and accurately diagnose infections and generate evidence for policy makers on the most cost-effective prevention and antimicrobial stewardship interventions, on how interventions should be implemented, and on policies that are also feasible in low-resource settings.

## Alignment with and complementarity to other AMR research agendas

In developing this research agenda, we collaborated with other relevant research initiatives to ensure complementarity and alignment, in addition to outlining in more detail the population, intervention or exposure, context, and comparator of interest, wherever possible. The One Health AMR research agenda was codeveloped by the Quadripartite^21^ comprising Food and Agriculture Organization, UN Environment Programme, WHO, and World Organisation for Animal Health, with the aim to improve knowledge on the intricate interactions of AMR among humans, animals, plants, and their shared environment. The WHO research agenda for AMR in human health includes crucial knowledge gaps that do not fall within the scope of the One Health agenda concerning the epidemiology, burden, and drivers of drug-resistant bacterial and fungal infections in the human health sector. The agenda also emphasises the development of optimised diagnostics and antimicrobial treatment strategies. The Strategic Research and Innovation research agenda from the future European Partnership on One Health AMR, co-funded by the member states and the European Commission under the Horizon Europe funding programme, is expected to guide future investments on AMR on topics encompassing both the One Health continuum and human health.^47^ Finally, this agenda aligns with the evidence gaps and needs identified by guideline development groups in the consolidated WHO guidelines on tuberculosis.^43^^,^^44^

## Strengths and limitations

We followed a transparent, reproducible, rigorous, and inclusive process.^48^ The metric-based CHNRI priority-setting approach minimised the potential bias stemming from the influence of a few individuals, a limitation commonly associated with consensus-based approaches.^23^ With the overall participation of 261 individuals from 69 countries (156 experts contributing to survey 1 and 234 experts contributing to survey 2), the agenda reflects a wide range of AMR expertise, geographical diversity, and representativeness, ensuring external relevance and validity of the outcomes. Yoshida and colleagues^49^ found that a sample size of 45–55 independent experts assigning a score on each question can ensure high reproducibility of the prioritisation results. Our study, with over 100 independent experts assigning a score on each topic, exceeded that threshold. Notably 40% offered perspectives exclusively from low-resource settings, underscoring the agenda’s attention to research topics pertinent to LMICs. By supplementing the list of topics identified by experts with a comprehensive scoping review spanning a 10-year period and capturing knowledge gaps in the existing literature, we are confident that we have included the most important topics and that those prioritised received strong agreement among experts. Notably, 74% of the 175 topics received an RPS of 0·80 or higher across all scoring criteria, affirming the validity of our approach. The research priorities included in the final list are broad and comprehensive. The research priorities effectively balance high-level concepts that capture the essence of multiple, sometimes overlapping, knowledge gaps and the more granular, focused research questions that could be used to design a study. Ultimately, each priority will require multiple studies of various types to be fully addressed, with potential variations based on country or setting.

Nonetheless, our project has some limitations. First, as experts were identified on the basis of their publication records, individuals from the WHO European region and region of the Americas were over-represented compared with those from other regions, reflecting a stronger investment in academic research in these two WHO regions. Second, assigning scores on research topics was tedious for the experts, as noted by some during the survey. Encouragingly, we did not observe a reduction in the number of experts participating in the scoring for topics included in the later part of the survey, suggesting that any potential survey fatigue did not negatively affect the results (data not shown). Third, the numbers of experts and topics varied across different AMR areas, which might have affected the final count of top research priorities in each area. However, simple scatter plots and correlation analyses did not show any obvious strong correlations between the number of participating experts for a given research topic in each AMR area and the final number of research topics from that AMR area in the 40 priorities (data not shown). In fact, experts were encouraged to assign scores on all topics they had knowledge of, without limiting themselves to their primary area of research expertise. Fourth, the scope of the agenda encompassed the WHO list of priority pathogens^4^ and therefore did not account for newly added^6^ and emerging pathogens and resistance mechanisms, some of which could be specific to the setting. Finally, developing a research agenda is, by its nature, a prioritisation exercise, resulting in only a subset of topics receiving the highest score. Many arguably important topics—although not feasible or prioritised within the proposed 2030 timeframe—still merit attention (appendix p 5). Examples include topics related to the effect of climate change on AMR in human health, determining the thresholds for clearing of colonisation by multidrug-resistant pathogens, and understanding the role of social norms (such as gender) in AMR awareness and individual and collective behaviour.^24^^,^^50^, ^51^, ^52^

## Implementation of the research agenda

WHO is committed to supporting the implementation of this agenda by focusing awareness around the 40 research priorities and fostering partnerships and collaboration with research funders and the global research community in the human health sector, including academia; partners such as WHO’s AMR collaborating centres network,^53^ Combating Antibiotic Resistant Bacteria Biopharmaceutical Accelerator,^54^ FIND,^55^ Global Antibiotic Research & Development Partnership,^56^ Global AMR R&D Hub,^46^ Global Strategy Lab’s The International Network for AMR Social Science,^57^ International Centre for Antimicrobial Resistance Solutions,^58^ and ReAct^59^; organisations such as the African Society for Laboratory Medicine, American Society for Microbiology, European Society of Clinical Microbiology and Infectious Diseases; funders and implementing partners including the AMR Action Fund, Bill & Melinda Gates Foundation, Centers for Disease Control and Prevention, European Commission-funded Joint programming initiative on AMR, Fleming Fund, Global Fund, National Institutions of Health, One Health Trust, Unitaid, Wellcome Trust, and World Bank; as well as governments.^10^ We urge all stakeholders to intensify investments and initiatives in AMR research, aligned with the priorities of the research agenda. A monitoring and evaluation framework will enable periodic assessment of the agenda’s effects in raising awareness, driving implementation, and seeking and influencing funding allocation.

## Conclusions

The WHO research agenda for AMR in human health comprises 40 research priorities that hold the potential to invigorate research efforts, generate evidence to improve existing treatment and diagnostic approaches, bolster the implementation of evidence-based national action plans for AMR, and ensure the feasibility of AMR interventions in low-resource settings. The research priorities reflect the most crucial knowledge gaps on AMR in human health that need to be addressed in the medium-to-long-term future (ie, by the year 2030) and aim to guide and encourage scientific interest and investment, generate evidence-based interventions to inform global and national health policies, and ultimately counteract the rise of AMR and its associated morbidity and mortality.

## Declaration of interests

SBe, ZD, CMC, HHB, NG, MH-A, BA, HS, TW, BDH, and VI are WHO employees. KVW and ACa were WHO employees. KUK and SBu are WHO consultants. ACh, DD, RLH, and IDO were WHO consultants. RLH is also supported by the Wellcome Trust (106680/Z/14/Z). All authors declare no other competing interests. Declarations of competing interests of the collaborators for the WHO research agenda for AMR in human health were reviewed and are available upon request to the corresponding author.
