# Supplementary material for: WHO global research priorities for antimicrobial resistance in human health
Source: Lancet Microbe. 2024 Nov;5(11):None. doi: 10.1016/S2666-5247(24)00134-4 (PMC11543637; doi:10.1016/S2666-5247(24)00134-4)
Supplement: Supplementary appendix [file mmc1.pdf]

THE LANCET
Microbe

Supplementary appendix

This appendix formed part of the original submission and has been peer reviewed.
We post it as supplied by the authors.

Supplement to: Bertagnolio S, Dobreva Z, Centner CM, et al. WHO global research priorities for antimicrobial resistance in human health. *Lancet Microbe* 2024. https://doi.org/10.1016/S2666-5247(24)00134-4

WHO Global Research Priorities for Antimicrobial Resistance in Human Health: Supplementary material

Table of contents

Supplementary material 1: Expert Group constitution and characteristics… Page 2

Supplementary material 2: Survey 1 spreadsheet… Page 5

Supplementary material 3: Survey 2 scoring spreadsheet… Page 5

Supplementary material 1: Expert Group constitution and characteristics

We aimed to include 300 experts in AMR and AMR-related fields, including experts in implementation research and experts in AMR policies and regulations. The identification of the experts was based on i) publication records in Web of Science and ii) recommendations.

The top authors publishing on AMR were identified through a structured search in Web of Science. The search focused on five cross-cutting areas of expertise and vertical areas of technical expertise related to AMR described below. This was to ensure that researchers in all fields related to the project were represented in the Expert Group. Once identified (see below methods for identifying and selecting members of the Expert Group), the group members were also asked to self-classify their professional background (e.g., researcher, programme manager).

The five overarching areas of expertise were:

1. Behavioural & social science
2. Health economics
3. Governance and regulations/legislation
4. Epidemiology
5. Pharmacological and clinical issues

The vertical areas of expertise were:

1. AMR burden and drivers
2. Antimicrobial stewardship
3. Antimicrobial use and consumption
4. Antimicrobials (including R&D)
5. Policies and regulations
6. AMR awareness and educations
7. IPC
8. WASH
9. Resistant tuberculosis
10. Immunization
11. Diagnosis/diagnostics
12. AMR foodborne diseases
13. AMR sexually transmitted diseases
14. Resistant fungi

Members of the expert group were identified based on their scientific production within each overarching and vertical area of expertise. Scientific production is defined as the number of peer-reviewed scientific publications within a 10-year set timeframe (2012-2021) identified through MeSH heading (descriptor) terms per cross-cutting and vertical areas of expertise, considering the scope of the project (Supplementary Tables 1 and 2). MeSH headings were chosen to reflect the scope of the research agenda setting project and those capturing specifically relevant terms and that include relevant terms within their tree structure. The search was done on the Medline database on the Web of Science platform between 27 December 2021 and 31 January 2022.

Authors were extracted from the results, searched for duplication (e.g. for alternative forms of author names) and the number of articles per author within each search was captured. The authors with the highest number of publications within each search were retained for further consideration.

A stepwise approach was used to identify the members of the expert group to ensure that 50% of experts were identified through the general search terms, 25% restricted to low- and middle-income countries (LMICs) and 25% having an institutional role in LMICs (Supplementary Table 3). The names identified through this approach were reviewed by the WHO steering group.

Supplementary Table 1: Overarching expertise search words in Web of Science for initial identification of members of Expert Group

Behavioural & Social science	MeSH terms:<br>Behavior or Behavior Observation Techniques or Adaptation, Psychological or Avoidance Learning or Applied Behavior Analysis or Behavior “and” Behavior

	Mechanisms or Behavior Control or Behavior Rating Scale or Behavior Therapy or Cooperative Behavior or Economics, Behavioral or Health Behavior or Helping Behavior or Imitative Behavior or Mass Behavior or Stereotyped Behavior or Verbal Behavior or Behavioral Research or Behavioral Medicine or Behavioral Sciences or Behavioral Disciplines “and” Activities or Social Behavior or Information Seeking Behavior or Drug-Seeking Behavior or Behavioral Risk Factor Surveillance System or Risk-Taking or Consumer Behavior or Professional Misconduct or Risk Reduction Behavior OR Social Environment or Social Participation or Social Behavior or Social Support or Social Change or Social Interaction or Reinforcement, Social or Social Adjustment or Social Conformity or Social Desirability or Social Medicine or Social Perception or Social Capital or Social Identification or Social Responsibility or Social Control, Formal or Social Control, Informal or Social Values or Sociological Factors or Social Learning or Social Factors or Hierarchy, Social or Social Sciences or Social Marketing or Social Vulnerability or Social Media or Social Control Policies or Social Norms or Social Theory or Socioeconomic Factors or Social Determinants of Health or Social Security or Social Planning or State Medicine or Psychosocial Support Systems
Health Economics	MeSH terms:<br>Models, Economic or Economic Development or Economic Factors or Economics, Hospital or Economics, Medical or Economics, Nursing or Economics, Behavioral or Economics or Value of Life or Cost of Illness or Cost-Benefit Analysis or Growth Health Care Economics “and” Organizations
Regulatory/Legislation	MeSH terms:<br>Legislation as Topic OR Legislation OR Government Regulation OR Jurisprudence OR Law Enforcement OR Mandatory Programs OR Patient Advocacy OR Policy Making OR Universal Health Care OR Social Control Policies
Epidemiology	MeSH terms:<br>Epidemiology OR Epidemiologic Factors OR Epidemiologic Methods OR Epidemiologic Measurements OR Disease Outbreaks OR Communicable Disease Control OR Population Surveillance OR Pharmacoepidemiology OR Epidemiological Models
Pharmacological (including access to Abs) and Clinical Issues	MeSH terms:<br>Pharmacoepidemiology OR Pharmacological “and” Toxicological Phenomena OR Pharmacology OR Drug Development OR Drug Discovery OR Pharmacologic Actions OR Clinical Competence OR Guideline Adherence OR Standard of Care OR Clinical Protocols OR Patient Care OR Patient Care Bundles OR Patient Care Management OR Clinical Studies as Topic OR Clinical Studies OR Diagnosis OR Clinical Nursing Research OR Chemistry, Clinical OR Clinical Medicine OR Ethics, Clinical OR Ethics Committees, Clinical OR Decision Support Systems, Clinical OR Clinical Decision Rules OR Diagnostic Services OR Practice Guideline OR Practice Guidelines as Topic OR Clinical Study OR Clinical Studies as Topic OR Laboratories, Clinical OR Quality Assurance, Health Care OR Delivery of Health Care OR Pharmaceutical Services OR Outcome Assessment, Health Care OR Medical Laboratory Personnel OR Medical Staff OR Nurses OR Nursing Staff OR Pharmacists OR Physicians OR Medical Informatics OR Biomarkers OR Patient Isolation

Supplementary Table 2: Vertical expertise search words in Web of Science for initial identification of members of Expert Group

General AMR	MESH terms (starting with Species=Humans):<br>Drug Resistance, Microbial OR Drug Resistance, Multiple NOT Virus Diseases NOT Viruses NOT HIV NOT HIV infections NOT HIV Protease NOT Viral Proteases NOT HIV Reverse Transcriptase NOT pol Gene Products, Human Immunodeficiency Virus NOT Antiviral Agents NOT HIV Integrase NOT env Gene Products, Human Immunodeficiency Virus NOT rev Gene Products, Human Immunodeficiency Virus NOT HIV Antigens NOT Viral Fusion Protein Inhibitors NOT HIV Long-Term Survivors NOT HIV Non-Progressors NOT HIV-Associated Lipodystrophy Syndrome NOT HIV Testing NOT Viral Load NOT Viral Pseudotyping NOT Virus Cultivation NOT HIV Envelope Protein gp41 NOT HIV Enhancer NOT HIV Wasting Syndrome NOT Viral Proteins NOT HIV Seroprevalence NOT HIV Serosorting NOT HIV Long Terminal Repeat NOT AIDS-Associated Nephropathy NOT AIDS Vaccines NOT HIV Enteropathy NOT tat Gene Products, Human Immunodeficiency Virus NOT Ribonuclease H, Human Immunodeficiency Virus NOT AIDS Dementia Complex NOT Receptors, HIV NOT AIDS-Related Opportunistic Infections NOT Lymphoma, AIDS-Related NOT AIDS Arteritis, Central Nervous System NOT AIDS Serodiagnosis NOT HIV Antibodies NOT gag Gene Products, Human Immunodeficiency Virus NOT pol Gene Products, Human Immunodeficiency Virus NOT AIDS-Related Complex NOT AIDS Arteritis, Central Nervous System NOT AIDS
1. AM stewardship and AMU	MeSH terms:<br>Antimicrobial Stewardship or Inappropriate Prescribing or Electronic Prescribing or Drug Prescriptions or Off-Label Use or Practice Patterns, Physicians' OR Drug utilization OR Professional Practice OR Guideline adherence OR Prescription drug misuse OR Anti-Infective Agents OR Guideline
2. AMR and AMU/AMC surveillance	MeSH terms:<br>Drug prescriptions OR Drug utilization OR Anti-infective agents OR Antibiotic prophylaxis OR Pharmacoepidemiology OR Benchmarking OR Health Surveys OR Population Surveillance OR Health Status Indicators OR Mass Screening
3. Antimicrobials	MeSH terms:

	(including R&D)	Drug Approval OR Drug Evaluation OR Drug Evaluation, Preclinical OR Product Surveillance, Postmarketing OR Anti-Infective Agents OR Pharmaceutical Research OR Drug Development OR Drug Discovery
4.	AMR Diagnostics	MeSH terms:<br>Diagnostic Techniques “and” Procedures OR Reagent Kits, Diagnostic OR Diagnostic Services OR Point-of-Care Testing OR Laboratories
5.	AMR National action plans (governance and implementation)	MeSH terms:<br>health policy OR health care reform OR national health policy OR policy making OR government programmes OR Government Publications as Topic OR Government Publication
6.	AMR awareness	MeSH terms:<br>Health Promotion OR Awareness OR Comprehension OR Consumer Health Information OR Health Fairs OR Patient Education as Topic
7.	IPC	MeSH terms:<br>Infection Control OR Hygiene OR Patient Isolation OR Infection Control Practitioners OR Universal Precautions OR Cross Infection
8.	WASH	MeSH terms:<br>Sanitation OR Sanitary Engineering OR Water Insecurity OR Water Microbiology OR Water Pollutants OR Water Resources OR Conservation of Water Resources OR Water Pollution OR Drinking OR Water Purification OR Waste Products OR Waterborne Diseases OR Sanitary Surveys, Water Supply OR Water Quality OR Disinfectants OR Hygiene
9.	Resistant tuberculosis	MeSH terms:<br>Tuberculosis, Multidrug-Resistant OR Tuberculosis Societies OR Antitubercular Agents
10.	Vaccines (including Product & Delivery Research)	MeSH terms:<br>Vaccines OR Vaccine-Preventable Diseases OR Vaccine Development OR Immunization Programs OR Immunization OR Vaccination Refusal
11.	AMR foodborne diseases	MeSH terms:<br>Foodborne Diseases OR Salmonella OR Salmonella Infections OR Shigella OR Dysentery, Bacillary OR Campylobacter Infections OR Campylobacter OR Staphylococcus OR Staphylococcal Infections OR Clostridium OR Clostridium Infections OR Escherichia coli OR Escherichia coli Infections OR Helicobacter pylori
12.	AMR sexually transmitted diseases	MeSH terms:<br>Sexually Transmitted Diseases, Bacterial OR Neisseria gonorrhoeae OR Gonorrhea

Supplementary Table 3: Terms used to identify researchers with affiliations in low- and middle-income countries

Terms used for restricting search to LMICs (added in the field “Address”)
afghanistan OR albania OR algeria OR american samoa OR angola OR "antigua and barbuda" OR antigua OR barbuda OR argentina OR armenia OR armenian OR aruba OR azerbaijan OR bahrain OR bangladesh OR barbados OR republic of belarus OR belarus OR byelarus OR belorussia OR byelorussian OR belize OR british honduras OR benin OR dahomey OR bhutan OR bolivia OR "bosnia and herzegovina" OR bosnia OR herzegovina OR botswana OR bechuanaland OR brazil OR brasil OR bulgaria OR burkina faso OR burkina fasso OR upper volta OR burundi OR urundi OR cabo verde OR cape verde OR cambodia OR kampuchea OR khmer republic OR cameroon OR cameron OR cameroun OR central african republic OR ubangi shari OR chad OR chile OR china OR colombia OR comoros OR comoro islands OR iles comores OR mayotte OR democratic republic of the congo OR democratic republic congo OR congo OR zaire OR costa rica OR "cote d'ivoire" OR "cote d' ivoire" OR cote divoire OR cote d ivoire OR ivory coast OR croatia OR cuba OR cyprus OR czech republic OR czechoslovakia OR djibouti OR french somaliland OR dominica OR dominican republic OR ecuador OR egypt OR united arab republic OR el salvador OR equatorial guinea OR spanish guinea OR eritrea OR estonia OR eswatini OR swaziland OR ethiopia OR fiji OR gabon OR gabonese republic OR gambia OR "georgia (republic)" OR georgian OR ghana OR gold coast OR gibraltar OR greece OR grenada OR guam OR guatemala OR guinea OR guinea bissau OR guyana OR british guiana OR haiti OR hispaniola OR honduras OR hungary OR india OR indonesia OR timor OR iran OR iraq OR isle of man OR jamaica OR jordan OR kazakhstan OR kazakh OR kenya OR "democratic people's republic of korea" OR republic of korea OR north korea OR south korea OR korea OR kosovo OR kyrgyzstan OR kirghizia OR kirgizstan OR kyrgyz republic OR kirghiz OR laos OR lao pdr OR "lao people's democratic republic" OR latvia OR lebanon OR lebanese republic OR lesotho OR basutoland OR liberia OR libya OR libyan arab jamahiriya OR lithuania OR macau OR macao OR republic of north macedonia OR macedonia OR madagascar OR malagasy republic OR malawi OR nyasaland OR malaysia OR malay federation OR Malaya federation OR maldives OR indian ocean islands OR indian ocean OR mali OR malta OR micronesia OR federated states of micronesia OR kiribati OR marshall islands OR nauru OR northern mariana islands OR palau OR tuvalu OR mauritania OR mauritius OR mexico OR moldova OR moldovian OR mongolia OR montenegro OR morocco OR ifni OR mozambique OR portuguese east africa OR myanmar OR burma OR namibia OR nepal OR netherlands antilles OR nicaragua OR niger OR nigeria OR oman OR muscat OR pakistan OR panama OR papua new guinea OR new guinea OR paraguay OR peru OR philippines OR philipines OR phillipines OR phillippines OR poland OR "polish people's republic" OR portugal OR portuguese republic OR puerto rico OR romania OR russia OR russian federation OR ussr OR soviet union OR union of soviet socialist republics OR rwanda OR ruanda OR samoa OR pacific islands OR polynesia OR samoan islands OR navigator island OR navigator islands OR "sao tome and principe" OR saudi arabia OR senegal OR serbia OR seychelles OR sierra leone OR slovakia OR slovak republic OR slovenia OR melanesia OR solomon island OR solomon islands OR norfolk island OR norfolk islands OR somalia OR south africa OR south sudan OR sri lanka OR ceylon OR "saint kitts and nevis" OR "st. kitts and nevis" OR saint lucia OR "st. lucia" OR "saint vincent and the grenadines" OR saint vincent OR "st. vincent" OR grenadines OR sudan OR suriname OR surinam OR dutch guiana OR netherlands guiana OR syria OR syrian arab republic OR tajikistan OR tadjikistan OR tadzhikistan OR tadzhik OR tanzania OR tanganyika OR thailand OR siam OR timor leste OR east timor OR togo OR togolese republic OR tonga OR "trinidad and tobago" OR trinidad OR tobago OR tunisia OR turkey OR turkmenistan OR turkmen OR uganda OR ukraine OR uruguay OR uzbekistan OR uzbek OR vanuatu OR new hebrides OR venezuela OR vietnam OR viet nam OR middle east OR west bank OR gaza OR palestine OR yemen OR yugoslavia OR zambia OR zimbabwe OR northern rhodesia OR global south OR africa south of the sahara OR sub-saharan africa OR subsaharan africa OR africa, central OR central africa OR africa, northern OR north africa OR northern africa OR magreb OR maghrib OR sahara OR africa, southern OR southern africa OR africa, eastern OR east africa OR eastern africa OR africa, western OR west africa OR western africa OR west indies OR indian ocean islands OR caribbean OR central america OR latin

america OR "south and central america" OR south america OR asia, central OR central asia OR asia, northern OR north asia OR northern asia OR asia, southeastern OR southeastern asia OR south eastern asia OR southeast asia OR south east asia OR asia, western OR western asia OR europe, eastern OR east europe OR eastern europe OR developing country OR developing countries OR developing nation? OR developing population? OR developing world OR less developed countr* OR less developed nation? OR less developed population? OR less developed world OR lesser developed countr* OR lesser developed nation? OR lesser developed population? OR lesser developed world OR under developed countr* OR under developed nation? OR under developed population? OR under developed world OR underdeveloped countr* OR underdeveloped nation? OR underdeveloped population? OR underdeveloped world OR middle income countr* OR middle income nation? OR middle income population? OR low income countr* OR low income nation? OR low income population? OR lower income countr* OR lower income nation? OR lower income population? OR underserved countr* OR underserved nation? OR underserved population? OR underserved world OR under served countr* OR under served nation? OR under served population? OR under served world OR deprived countr* OR deprived nation? OR deprived population? OR deprived world OR poor countr* OR poor nation? OR poor population? OR poor world OR poorer countr* OR poorer nation? OR poorer population? OR poorer world OR developing econom* OR less developed econom* OR lesser developed econom* OR under developed econom* OR underdeveloped econom* OR middle income econom* OR low income econom* OR lower income econom* OR low gdp OR low gnp OR low gross domestic OR low gross national OR lower gdp OR lower gnp OR lower gross domestic OR lower gross national OR lmic OR lmics OR third world OR lami countr* OR transitional countr* OR emerging economies OR emerging nation?

This process yielded a list of 125 experts.

We then approached members of the WHO Research Agenda for AMR in Human Health steering group, the WHO Strategic Advisory Group on AMR, and WHO regional advisers to identify additional experts, particularly those from low- and middle-income countries, aiming to ensure gender balance. We also enriched for expertise in paediatrics, mycology, food-borne diseases, WASH, immunisation, implementation research, AMR policy and regulations, and program management (particularly in the context of health ministries). The decision to include a recommended expert was based on a qualitative evaluation of the expert's country, expertise, affiliation, and research output (volume and appropriateness). This process was also undertaken to increase the number of participating experts in survey 2.

Supplementary material 2: Survey 1 spreadsheet

Excel spreadsheet: Survey 1

Supplementary material 3: Survey 2 scoring spreadsheet

Excel spreadsheet: Survey 2
